# Flipping the classroom in neurological bedside teaching: a prospective controlled study

**DOI:** 10.1186/s12909-023-04150-2

**Published:** 2023-03-15

**Authors:** Henrik Heitmann, Elisabeth Fischer, Philipp Wagner, Dennis Pötter, Martin Gartmeier, Friederike Schmidt-Graf

**Affiliations:** 1 Department of Neurology, School of Medicine, Technical University of Munich (TUM), Munich, Germany; 2 Department of Psychosomatic Medicine and Psychotherapy, Technical University of Munich (TUM), Munich, Germany; 3 Department of Anesthesiology and Operative Intensive Care Medicine, Klinikum Dortmund, Dortmund, Germany; 4 TUM Medical Education Center, School of Medicine, Technical University of Munich (TUM), Munich, Germany

**Keywords:** Bedside teaching, Clinical competence, Neurology

## Abstract

**Background:**

Bedside teaching is essential to foster core clinical competences in medical education, especially in Neurology. However, bedside skills are declining and new concepts to enhance the effectiveness of bedside teaching are needed, also in view of limited in-person teaching possibilities in the ongoing pandemic situation. If theoretical knowledge is taught prior to in-person sessions this might allow to better focus on practical application aspects during bedside teaching. We thus aimed to answer the question to what extent such an approach can enhance the effectiveness of neurological bedside teaching.

**Methods:**

In this prospective controlled study, neurological bedside courses following a traditional and a flipped classroom (FC) approach were compared with regards to their effects on theoretical knowledge and practical skills of medical students. Evaluations were obtained from 161 students and their lecturers participating in a neurological bedside teaching course at a German university hospital between October 2020 and July 2021. Students were randomly assigned to course dates. However, the 74 students assigned to course dates from May to July 2021 completed a mandatory online preparation course prior to the bedside teaching. These students served as the interventional group (IG) and the remaining 87 students formed the control group (CG). Ratings of knowledge and skills provided by the students and their lecturers on numerical rating scales served as primary outcome measures. Moreover, the time needed to recapitulate theoretical contents during the in-person teaching session was assessed as a secondary outcome measure. Group comparisons were performed using t-statistics.

**Results:**

Theoretical knowledge upon entering the course was rated significantly higher in the IG by the students (*p* < 0.001) and lecturers (*p* = 0.003). Lecturers also rated the practical skills of students in the IG significantly higher (*p* < 0.001). Furthermore, significantly less time was needed to recapitulate theoretical contents during the in-person session in the IG (*p* = 0.03).

**Conclusions:**

Using a FC approach enhances the effectiveness of in-person neurological bedside teaching. Thus, these concepts are particularly valuable in the ongoing pandemic situation. Moreover, they might allow to reuse e-learning contents developed during the pandemic and to develop future bedside teaching concepts.

## Introduction

Bedside teaching is an essential part of medical education with a fundamental importance in fostering the case-based application of theoretical knowledge and acquiring of practical skills, including hypothesis-driven examination techniques and clinical reasoning [1, 2]. These aspects are particularly important for teaching Neurology and the Neurological Exam (NE) [3]. However, there are several challenges that need to be met. First, the NE is largely perceived as difficult by medical students, thereby fostering a phenomenon that has been termed “Neurophobia” [4, 5]. Second, bedside and examination skills have been constantly decreasing over the past decades [1, 2], especially in the field of Neurology [6]. Third, the present pandemic situation has largely aggravated these problems due to suspension of clinical placements including bedside teaching in Neurology [7–9] and beyond [1, 10]. In view of these challenges, new concepts for in-person bedside teaching are urgently needed [1]. In this context, blended learning approaches might be of particular interest for the development of future clinical teaching formats [11, 12]. There is mounting evidence that such approaches combining e.g. e-learning materials with face-to-face instruction sessions are more effective than traditional formats, especially in medical education [13, 14]. Particularly, flipped classroom approaches (FC), are increasingly being applied in the health professions [15, 16]. The term flipped classroom refers to the general concept that in contrast to traditional teaching formats “foundational knowledge is gained by students through self-paced learning prior to class. Knowledge application and problem solving then occur inside the classroom through instructor-facilitated learner-centered activities” [16]. This approach has been shown to improve the transfer of theoretical knowledge into practical application as well as to enhance learner engagement in medical education [15–17]. The rather broad general definition of the concept allows for considerable variability of implementations in different teaching settings [16]. The FC approach has been largely applied for medical lectures [15], also in the field of Neurology [18]. However, studies examining this approach in practically oriented neurological teaching formats are scarce [19]. Thus, additional evidence for its effectiveness in this context is needed, also in view of the substantial initial expenditures necessary to implement such a concept [19]. A wide range of materials is used for preparation in FC concepts, with videos and animated presentations being most widely used and well-received by the students [15, 16], Interestingly, the pandemic situation has led to the creation of many such teaching videos and animated presentations used as e-learning contents to replace and complement practically-oriented clinical placements [11, 20], also in the field of Neurology [9, 21, 22]. Thus, re-using this e-learning content to implement FC concepts also for practical formats including bedside teaching might be particularly effective [12]. We hypothesized that using e-learning materials in a FC approach – including content developed during the pandemic – enhances the effectiveness of neurological bedside teaching. More specifically, the aim of the present study was to evaluate to what extent enhancing theoretical knowledge beforehand enables students to better focus on and acquire practical applications during in-person bedside teaching. To this end, we compared a traditional and a FC neurological bedside teaching format regarding their effects on medical students’ competences, i.e. their theoretical knowledge and their ability to perform and interpret a NE.

## Methods

### Participants and study design

**Fig. 1:**
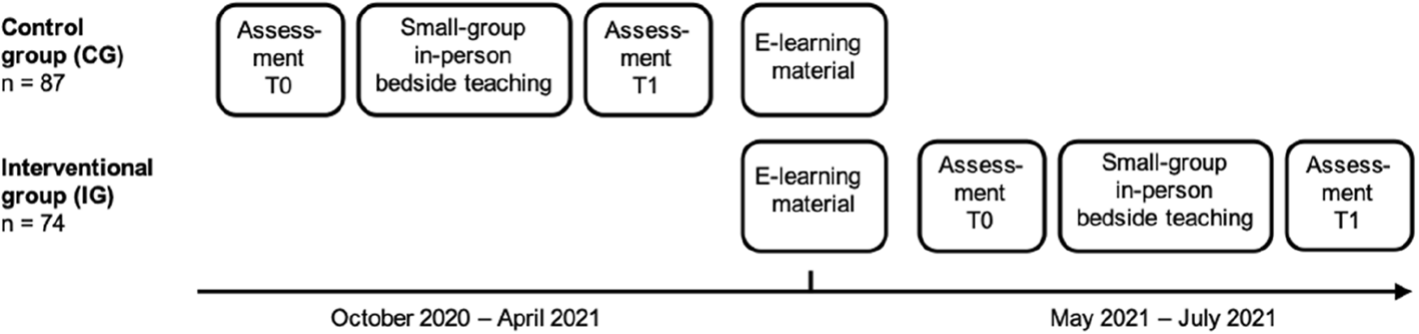
Study design. The e-learning material was made available to students in the control group in May 2021, thus after the course, whereas the interventional group had to complete the e-learning material in the context of a mandatory online preparation course prior to participation in the in-person teaching session

All medical students scheduled to participate in a non-elective neurological bedside teaching course with patient contact at TUM School of Medicine in Munich, Germany between October 2020 and July 2021 (*n* = 174) were eligible for inclusion into the study. There were no further exclusion criteria. Randomized assignment of students to course dates was performed by the TUM Medical Education Center. Students assigned to course dates from May to July 2021 (*n* = 82) had to complete a mandatory online preparation course prior to in-person teaching. These students formed the interventional group (IG). The remaining students assigned to course dates in October 2020 and April 2021 (*n* = 92) participated in a traditional course format with in-person teaching only and therefore served as the control group (CG). For the latter students the online course with all e-learning materials was made available after their participation in the in-person teaching session. All students were asked to voluntarily evaluate the course and subsequently a total of 161/174 students (87/92 in the CG and 74/82 in the IG) anonymously completed the corresponding evaluation forms at the beginning and end of the in-person teaching session (see Fig. 1). A part of the CG in the present study also formed the CG in a previous study with overlapping methodology that compared bedside teaching approaches with and without patient contact during the COVID-19 pandemic [12].

The present study had a prospective controlled design. It was approved by the ethics committee of the Medical Faculty of the Technical University of Munich (TUM) and performed in accordance with the relevant guidelines and regulations. For obtaining the voluntary and anonymous evaluations informed consent was waivered by the ethics committee.

### Bedside teaching

The neurological bedside teaching at TUM School of Medicine takes place in the fourth year, which is the students’ second year of clinical training. In advance, all students receive a script providing background information on the performance and clinical interpretation of the NE to prepare for the course. Subsequently, students attend an in-person teaching session in small groups of maximum three students per lecturer for three hours on one afternoon. Lecturers are resident physicians at the TUM Department of Neurology. At the beginning of the course theoretical contents are recapitulated as needed. To this end, lecturers give a short introduction on the theoretical backgrounds of the NE. This includes a repetition of functional neuroanatomy basics and their implications for principles of the NE, e.g. the importance of symmetry. Moreover, students are provided with a structured approach on how to perform, interpret and report the NE. Additionally, the importance of history taking in guiding the clinical examination procedure is emphasized. After that, the lecturers demonstrate the performance of the NE and students can practice on their peers. Subsequently, students go to the bedside to perform history taking and a full NE on patients. Afterwards, students present the patients cases to the lecturers and pathological findings in the NE are re-assessed together.

### Online preparation course (e-learning)

In addition to the script provided to both groups, students in the IG were invited to additionally complete a mandatory online preparation course one week prior to their in-person teaching session. As proof of successful completion students received a certificate after correctly answering a set of multiple-choice questions on the course contents. Besides the script, that was also available to the CG, the online preparation course comprised a video and a screencast on the NE that were developed and produced together with the TUM Medical Education Center. The 12-min long video shows how to perform a neurological screening exam, focusing on relevant core components in the evaluation of gait, coordination, cranial nerves, muscle strength, sensation and reflexes [23]. The 21-min long screencast provides additional information on the theoretical backgrounds and practical advice to the performance of the different parts of the NE. Theoretical backgrounds include aspects of functional neuroanatomy and basic principles of the NE such as the importance of symmetry. Practical advice was focused on aspects that had been perceived as challenging by the students in previous years and included short video sequences, e.g. on what to do if reflexes are difficult to obtain.

### Assessment

Evaluations were obtained at two different time points, before (T0) and after the course (T1), as previously reported [12].

#### Student ratings

Students were asked to rate different aspects of their theoretical knowledge and their practical skills regarding the performance and interpretation of the NE, using identical evaluation forms at T0 and T1. Ratings were obtained using numerical rating scales (NRS) from 0 (“not good at all”) to 10 (“very good”). For the theoretical knowledge, items covered were *functional neuroanatomy* (e.g. knowing the names of the cranial nerves and their physiological function; being able to differentiate upper and lower motor neuron signs), *systematology* (e.g. knowing into which parts the NE can be divided and the best sequence to assess them), *basic principles* (e.g. knowing the importance of symmetry when performing a NE and how to differentiate physiological from pathological findings) as well as *quantification* (e.g. knowing how to grade muscle strength or reflexes). Items regarding practical skills were *examination skills* (e.g. ability to autonomously perform a neurological screening exam), *information transfer* (e.g. ability to use information derived from history taking to focus the examination procedure), *documentation* (e.g. ability to document findings in a structured manner), *oral presentation* (e.g. ability to communicate findings in a structured manner), and *red flags* (e.g. ability to identify alarming signs in the NE). Overall scores for theoretical knowledge and practical skills were obtained by averaging the respective single item scores mentioned above. Furthermore, students could provide free-text comments including suggestions for improvement at T1. Additionally, students in the IG were asked to rate their agreement with statements on the FC teaching concept on a NRS from 1 (“do not agree at all”) to 5 (“fully agree”) at T1. Example statements were:

- A. “The teaching concept with e-learning preparation material contributes to a better understanding of the subject matter.”
- B. “Important topics, procedures and techniques are more easily accessible through the combination of e-learning preparation material and in-person practical application.”
- C. “The e-learning preparation material allowed for a better focus on learning and applying practical skills during the in-person teaching session.”

#### Lecturer ratings

Lecturers were asked to rate the average level of their students’ group (i.e. the subjective mean across their small group of maximum three students) regarding prior theoretical knowledge as well as the practical skills acquired, taking into account the previously mentioned criteria, from 0 (“not good at all”) to 10 (“very good”) on an NRS at T1. Additionally, lecturers registered the time (in minutes) needed to recapitulate theoretical contents with the students at the beginning of the course before going bedside.

### Statistics

Statistical analyses were performed using JASP (JASP Team 2021, Version 0.15). To compare student and lecturer ratings between the IG and the CG student t-tests for independent samples, including estimates of effect size (Cohen’s d) and the respective 95% confidence intervals, were used. Values of d = 0.2, 0.5 and 0.8 were considered indicative of small, medium and large effect sizes, respectively. A sensitivity analysis for independent sample t-statistics using G*Power [24] showed that the present sample size was sufficient to detect potential group differences with medium effect sizes (Cohen’s d = 0.45), given an error probability (α) of 0.05 and a power of 0.8. The significance level for all statistical tests was set to 0.05 two-tailed.

### Availability of data and materials

The datasets used and/or analysed during the current study are available from the corresponding author on reasonable request.

## Results

### Self-ratings of knowledge and skills by the students

Students rated their respective theoretical knowledge and their practical skills regarding different aspects of the neurological exam at the beginning (T0) and end (T1) of the course.

**Fig. 2:**
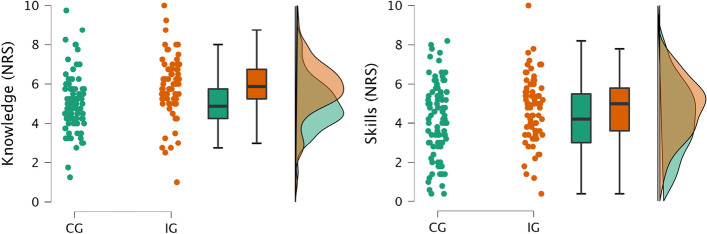
Comparison of averaged student self-ratings of theoretical knowledge and practical skills at T0. Raincloud plots show individual values as well as boxplots and probability maps. NRS = Numerical Rating Scale, CG = control group, IG = interventional group

At T0 average ratings for knowledge (t = -3.7, *p* < 0.001, Cohen’s d = -0.58) and skills (t = -2.2, *p* = 0.03, Cohen’s d = -0.35) were significantly higher in the IG compared to the CG, with medium and small effect sizes respectively (see Fig. 2). No group differences were obtained for average ratings at T1. However, students in the IG rated their examination skills significantly higher compared to the CG (t = -2.0, *p* = 0.04, Cohen’s d = -0.32, for detailed results including single items and test statistics please see Table 1).

**Table 1:** Students’ self-ratings of knowledge and skills

	**Items**	**Baseline T0** *(mean* ± *SD)*		*Independent sample t-test*	**Follow-up T1** *(mean* ± *SD)*		*Independent samplet-test*
		***CG***	***IG***	***CG vs. IG***	***CG***	***IG***	***CG vs. IG***
**Know-ledge**	*Functional neuroanatomy*	6.2 ± 1.5	6.5 ± 1.7	t_df (159)_ = -1.3 *p* = 0.20	8.0 ± 1.3	8.2 ± 1.3	t_df (159)_ = -0.8 *p* = 0.40
	*Systematology*	5.1 ± 2.0	5.8 ± 1.7	t_df (159)_ = -2.3 *p* = 0.02	8.5 ± 1.2	8.6 ± 1.2	t_df (159)_ = -0.2 *p* = 0.83
	*Basic principles*	5.0 ± 1.8	6.2 ± 1.9	t_df (159)_ = -4.1 *p* < 0.001	8.4 ± 1.1	8.2 ± 1.3	t_df (159)_ = 1.2 *p* = 0.23
	*Quantification*	3.7 ± 2.1	4.9 ± 2.2	t_df (159)_ = -3.6 *p* < 0.001	7.5 ± 1.7	7.8 ± 1.7	t_df (159)_ = -0.8 *p* = 0.42
	***Average***	5.0 ± 1.5	5.9 ± 1.5	t_df (159)_ = -3.7 *p* < 0.001 d = -0.58 CI = [-0.90, -0.26]	8.1 ± 1.1	8.2 ± 1.2	t_df (159)_ = -0.3 *p* = 0.78 d = -0.05 CI = [-0.36, 0.27]
**Skills**	*Examination*	3.4 ± 2.3	4.3 ± 1.9	t_df (159)_ = -2.6 *p* = 0.01	7.6 ± 1.4	8.0 ± 1.1	t_df (158)_ = -2.0 *p* = 0.04
	*Information transfer*	4.8 ± 2.1	5.2 ± 1.8	t_df (159)_ = -1.3 *p* = 0.21	7.7 ± 2.2	7.7 ± 1.4	t_df (158)_ = 0.2 *p* = 0.83
	*Documentation*	4.1 ± 2.2	4.4 ± 2.6	t_df (159)_ = -1.1 *p* = 0.29	6.6 ± 1.5	6.8 ± 1.8	t_df (158)_ = -0.7 *p* = 0.47
	*Oral presentation*	4.1 ± 2.2	4.7 ± 1.8	t_df (159)_ = -1.9 *p* = 0.06	6.9 ± 1.5	7.1 ± 1.7	t_df (158)_ = -0.9 *p* = 0.35
	*Red flags*	4.3 ± 2.2	5.3 ± 2.2	t_df (159)_ = -2.7 *p* = 0.01	7.3 ± 1.5	7.6 ± 1.5	t_df (158)_ = -1.1 *p* = 0.26
	***Average***	4.2 ± 1.7	4.8 ± 1.2	t_df (159)_ = -2.2 *p* = 0.03 d = -0.35, CI = [-0.66, 0.04]	7.2 ± 1.2	7.4 ± 1.2	t_df (159)_ = -1.1 *p* = 0.27 d = -0.18 CI = [-0.49, 0.14]

The table shows the students’ self-ratings of knowledge and skills on group level including the results from independent sample t-tests to assess differences between groups at T0 and T1. *CG* Control group, *IG* Interventional group, *SD* Standard deviation, *T0* Baseline assessment before the course, *T1* Follow-up assessment after the course, *d* Cohen’s d, *CI* 95% confidence interval

### Ratings of knowledge and skills by the lecturers

**Fig. 3:**
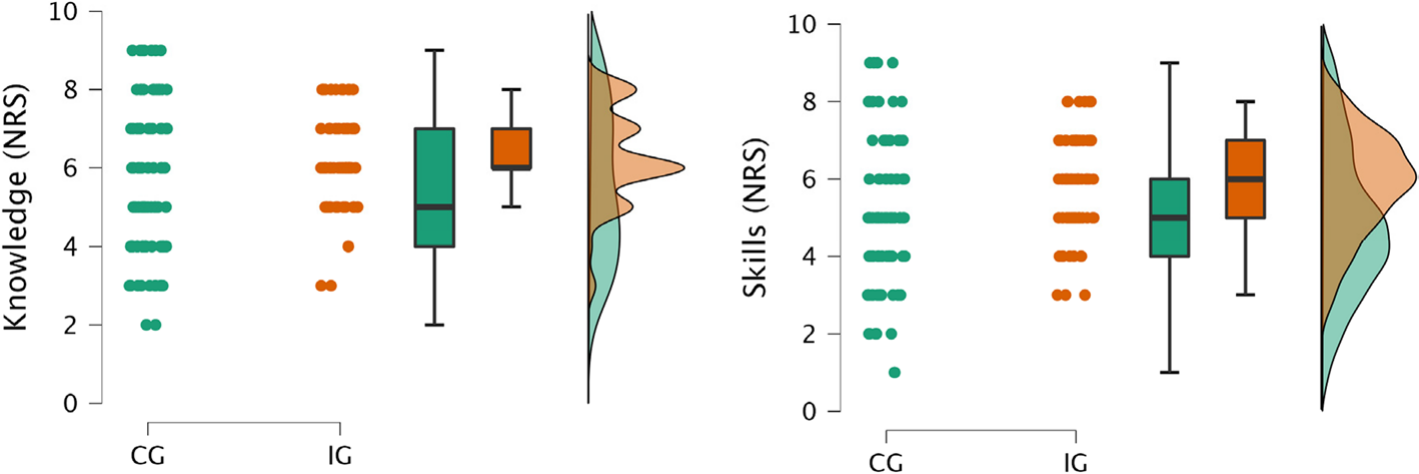
Comparison of lecturer ratings of theoretical knowledge and practical skills. Lecturers rated the students’ previous theoretical knowledge upon entering the course as well as the practical skills obtained during the course on a numerical rating scale (NRS) from 0–10 at T1. Raincloud plots show individual values as well as boxplots and probability maps. CG = control group, IG = interventional group

Lecturers were asked to provide average ratings of previous theoretical knowledge and the skills acquired during the course on group level for their respective small group of students. Ratings of knowledge (mean ± SD 5.6 ± 2.3 vs. 6.3 ± 1.2; t = -3.0, *p* = 0.003, Cohen’s d = -0.45) and skills (mean ± SD 4.9 ± 1.9 vs. 5.9 ± 1.3; t = -3.7, *p* < 0.001, Cohen’s d = -0.60) were significantly higher and showed a much lower standard deviation in the IG compared to the CG, with medium effect sizes respectively (see Fig. 3).

### Time needed to recapitulate theoretical knowledge

**Fig. 4:**
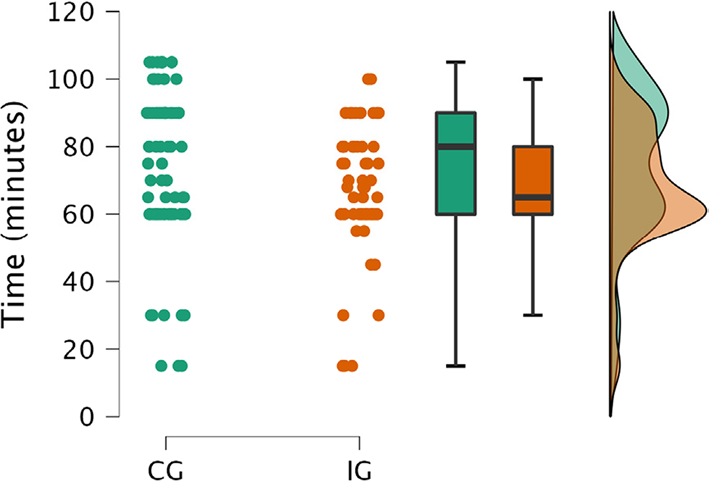
Comparison of time (minutes) needed to recapitulate theoretical contents during the in-person teaching session before going bedside. Raincloud plots show individual values as well as boxplots and probability maps. CG = control croup, IG = interventional group

Lecturers were asked to register the time they needed to recapitulate theoretical knowledge regarding the NE before being able to go bedside for practical teaching during the in-person teaching session. Significantly less time of the total course duration of 180 min was needed to recapitulate theoretical contents in the IG compared to the CG (mean ± SD 74 ± 22 vs. 67 ± 18; t = 2.3, *p* = 0.02, Cohen`s d = 0.36) (see Fig. 4).

### Students´ free-text comments and agreement ratings for the FC concept

Several students in the CG commented that they were grateful to be able to participate in an in-person teaching format despite the pandemic situation. However, students stated that in view of their very limited prior experience with the NE, the course time was perceived as not sufficient to gain proficiency and to comfortably perform and interpret the NE. In this context students explicitly suggested the use of further preparation materials, especially videos showing the examination procedure. Correspondingly, several students in the IG commented that the online preparation material was perceived as very helpful. This was also reflected by high agreement scores for the new teaching concept. Mean scores ± SD on the NRS from 1 to 5 for Statement A (“contributes to a better understanding of the subject matter”), Statement B (“procedures and techniques are more easily accessible”) and Statement C (“allows for a better focus on learning and applying practical skills during the in-person teaching”) were 4.2 ± 0.89, 4.3 ± 0.87 and 4.3 ± 0.82, respectively.

## Discussion

In the present study, student and lecturer ratings of theoretical knowledge and practical skills for a traditional and a FC neurological bedside teaching format were assessed and compared. Ratings of students and lecturers revealed a significantly higher and more homogenous level of theoretical knowledge upon entering the in-person teaching session in the IG that used blended learning. Additionally, lecturers rated the practical skills obtained during the course significantly higher in the IG. Moreover, less time was needed to recapitulate theoretical contents at the beginning of the course in this group. However, students’ average self-ratings of theoretical knowledge and practical skills, apart from examination skills, after the course did not differ between the two groups. Acceptance of the FC concept among students was high.

The finding of a higher and more homogenous entrance level of knowledge and skills in the group using a FC approach shows that the e-learning materials provided were effective in preparing the students for the in-person teaching session. An effective transfer of theoretical knowledge prior to participating in face-to-face teaching sessions was previously identified as one of the key mechanisms for successful blended learning approaches in medical education [13–15]. The higher overall skill-ratings by the lecturers and the higher self-ratings for examination skills by the students at T1 in the IG further contribute to the mounting evidence, that a FC approach enhances the effectiveness of teaching practical applications [15, 25]. Moreover, our findings are in line with results from a recent study, showing that blended learning approaches using videos are particularly suited to teach neurological examination skills, as assessed with an objective structured clinical examination (OSCE) [26]. The present study complements these findings by showing similar effects on subjective ratings by the students and lecturers including the levels of knowledge and skills upon entering the course. Furthermore, it extends these findings to the setting of neurological bedside teaching where the focus is not only on acquiring examination techniques but rather on applying them to real patient cases [1]. However, diverging from the lecturers’ ratings the students’ self-ratings of knowledge and particularly skills, apart from examination skills, did not differ between groups at T1. This might be attributed to the type of e-learning materials provided for preparation that had a clear focus on examination techniques and might thus have less influence on other aspects of bedside teaching. The lack of a difference might also be partially explained by a ceiling effect, since the students’ ratings of skills and knowledge at T1 were also considerably high in the CG. These high ratings in the CG might be driven by a pandemic effect. This notion is supported by the free-text comments provided by the students, where they explicitly expressed their gratefulness for being able to participate in a practical in-person teaching session with patient contact despite the ongoing pandemic, which might be reflected by higher ratings. This is in line with previous findings by the authors [12] and a recent review on replacement teaching formats during the COVID-19 pandemic [27].

The other main finding was that in the IG significantly less time was needed to recapitulate theoretical contents during the in-person teaching session. Thus, more time of the course could be spent bedside. This time-saving effect again complements results from the previously mentioned study by Bornkamm and colleagues, where students in the blended learning group showed better examination skills despite having less face-to-face course time [26]. Hence, such concepts might be particularly valuable in the present pandemic situation, where due to limited time-windows and personnel for in-person teaching an effective preparation and a focus on practical applications are even more critical [1, 12]. In line with previous studies student’s acceptance and agreement with potential advantages of the FC concept was high [15, 16].

Several limitations have to be taken into account when interpreting the present results. First, the present findings are largely based on subjective ratings and not on standardized performance assessments. However, student feedback is a valuable source of information [28] which does relate to the clinical performance in the objective assessment of neurological examination skills [29]. Moreover, the very nature of these evaluations provides insights on the students’ and lecturers subjective experiences in these challenging times. Second, the important aspect of potential group differences in learner engagement have not been formally assessed in the present study. The students’ free text-comments and agreement ratings for the FC concept might allow to partially infer general notions in these regards but future studies should use formal assessments to capture the different facets of learner engagement post blended learning materials and include them as outcome parameters [30]. Third, since the study design did not allow for blinding, results might be partially driven by expectation and novelty effects of lecturers and students respectively. Additionally, familiarity with the hybrid course scenario in general might have played a role on the learners’ side. Fourth, the effects observed might be partially attributable to the higher amount of preparation materials provided to the IG per se. However, potential effects of such an intensified preparation schedule on the structure of the following in-person teaching session were captured by assessing the time needed to recapitulate theoretical contents as a secondary outcome measure. Fifth, data acquisition had to be paused between November 2020 and March 2021 due to contact restrictions. Thus, the data of the CG were obtained during two consecutive semesters and not, as initially planned, during one semester.

## Conclusions

In conclusion, using a FC approach enhances the effectiveness of neurological bedside teaching by improving theoretical knowledge beforehand, so that in-person teaching can better focus on practical applications. Thus, the effective transfer of knowledge into practice is fostered, especially regarding examination skills. Moreover, this approach not only holds the potential to save resources but also increases patient safety in the present pandemic situation. Additionally, these concepts might allow to reuse and refine e-learning contents developed during the pandemic to shape the future of bedside teaching beyond emergency remote teaching.

## Data Availability

The datasets used and/or analysed during the current study are available from the corresponding author (HH) on reasonable request.

## Abbreviations

CI: Confidence interval
FC: Flipped classroom
CG: Control group
IG: Interventional group
NRS: Numerical rating scale
SD: Standard deviation
T0: Baseline evaluation before the course
T1: Follow-up evaluation after the course
TUM: Technical University of Munich
